# Ectopic thyroid gland located on the L4 vertebral body: A case report

**DOI:** 10.1097/MD.0000000000024042

**Published:** 2021-01-15

**Authors:** Qihuan Lin, Qilu Gao, Rong Fan, Li Zhang, Weijia Li, Hongkun Liu, Wenbin Zheng

**Affiliations:** Department of Radiology, The Second Affiliated Hospital, Medical College of Shantou University.

**Keywords:** ectopic thyroid, lumbar spine, magnetic resonance imaging, PET/ computed tomography

## Abstract

**Rationale:**

The prevalence of ectopic thyroid is extremely low, with the condition observed in approximately 1 in 100,000 to 300,000 people. Thyroid gland ectopia develops as a result of the presence of developmental abnormalities during the migration of the thyroid anlage from the floor of the primitive foregut to its final position in the neck. Ectopic thyroid tissue is commonly observed in the lingual region, but can also present in other head and neck regions, as well as regions located at a large distance from the neck.

**Patient concerns:**

A 67-year-old woman who had experienced left lumbago and leg pain was transferred to our hospital following the worsening of her lumbago-related symptoms in the 2 months preceding her presentation. Seven years ago, the patient had recurrent lumbago and leg pain without obvious inducement, and visited a local clinic for treatment. The severity of her symptoms fluctuated; their intensity increased after participation in activities and decreased after rest.

**Diagnoses:**

The patient was diagnosed as having an ectopic thyroid gland that was located on the L4 vertebral body.

**Interventions:**

The patient chose to undergo surgery, with supportive care, following tumor discovery.

**Outcomes:**

After surgical treatment, the degree of lumbar spinal stenosis improved, and the patient's clinical symptoms were alleviated.

**Lessons:**

Clinically, ectopic goiter is diagnosed through radionuclide thyroid imaging, ultrasound examination, computed tomography, magnetic resonance imaging, and biopsy pathology. However, the imaging manifestations in this case were atypical, leading to greater diagnostic difficulties. A conclusion was finally reached based on pathology.

## 1 Introduction

The incidence of ectopic thyroid is extremely low. The condition presents in only approximately 1 in 100,000 to 300,000 people, and 1 in 4000 to 8000 people with thyroid disease have an ectopic thyroid.^[2]^ Thyroid gland ectopia develops as a result of developmental abnormalities that are present during the migration of the thyroid anlage from the floor of the primitive foregut to its final position in the neck.^[3]^ Ectopic thyroid tissue is commonly observed in the lingual region, but can be detected in other head and neck regions too,^[1]^ including the trachea, submandibular region, lateral cervical region, armpit, palatine tonsil, carotid bifurcation, iris, and pituitary gland. In addition, ectopic thyroid tissue has also been identified in regions located at a large distance from the neck, including the heart, ascending aorta, thymus, esophagus, duodenum, gallbladder, gastric bed, pancreas, intestinal stroma, hepatic portal, adrenal gland, ovary, fallopian tube, uterus, and vagina.^[2]^ This article presents a rare case of a 67-year-old woman with left lumbago and leg pain, in whom appropriate diagnoses and treatment led to positive outcomes.

## 2 Case report

### 2.1 Clinical data

A 67-year-old woman who had experienced left lumbago and leg pain for 7 years was transferred to our hospital. Seven years prior to the presentation, the patient had experienced recurrent lumbago and leg pain without obvious inducement, and visited a local clinic for treatment. The symptoms fluctuated; their intensity increased after participation in activities and decreased after rest. She was admitted to a local hospital following an increase in the severity of her lumbago-related symptoms in the last 2 months. After treatment, no significant improvements were observed. The patient then visited the outpatient department of our hospital for an examination. A previous history of bronchial asthma, hypertension, and type 2 diabetes was recorded. The patient underwent surgical treatment for the removal of a tumor that was located on the lumbar vertebral body. Bilateral thyroidectomy was performed with the patient's consent, as the presence of metastasis could not be ruled out by pathology. After the surgery, the patient's physical condition gradually improved and the severity of her clinical symptoms decreased.

The levels of tumor markers, including carcinoembryonic antigen, alpha-fetoprotein, and CA-125 were all within the normal range. However, the CA-199 level was 42.69 U/mL (normal value: 0–37 U/mL). Laboratory thyroid function tests were not performed before the surgery.

Thyroid function examinations, performed following the lumbar focus resection, showed the following results: total thyroxine: 139.00 μg/L (normal range: 54.4–118.5 μg/L), total triiodothyronine: 1.48 μg/L (normal range: 0.66–1.61 μg/L), free triiodothyronine: 5.79 pmol/L (normal range: 3.29–6.47 pmol/L), free thyroxine: 13.19 pmol/L/l (normal range: 7.64–16.03 pmol/L), thyroid-stimulating hormone: 0.05 mIU/l (normal range: 0.49–4.91 mIU/L), thyroglobulin antibodies: 30.90 IU/ml (normal range: 0–60 IU/ml), and anti-thyroid peroxidase (TPO): <28.0 IU/ml (normal range: 0–60 IU/ml).

### 2.2 Imaging data

#### 2.2.1 Scan parameters

A. Computed tomography (CT): A 64-slice spiral CT scanner was used for the examination, which was conducted with the patient in a supine position. Scanning was performed in the axial plane. The scanning conditions were as follows: tube current, 400 mA; tube voltage, 120 kV, layer thickness, 0.625 mm; interval, 1 mm; and scanning type, spiral.
B. Magnetic resonance imaging (MRI): T1 weighted image (T1WI): repetition time (TR) 2497 ms, echo time (TE) 6.85 ms; T2 weighted image (T2WI): TR 2900 ms, TE 124.12 mm; fat-suppressed T2: TR 3200 ms, TE 121.68 ms, layer thickness: 5 mm, layer spacing: 1 mm, field of view 32 cm×32 cm, matrix: 384 × 192, and number of excitations: 4.

#### 2.2.2 Imaging results

**Figure 1:**
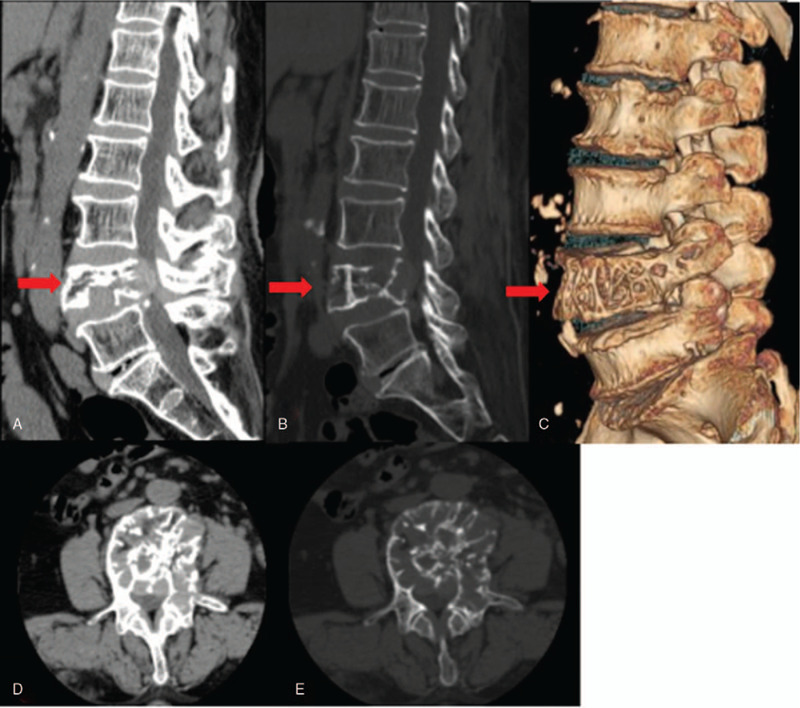
(A–E). Bone resorption and destruction changes as observed in the L4 vertebral body and left pedicle, showing a sparse bone crest structure in the L4 vertebral body and palisade-like changes. A mass-like soft tissue shadow with a size of about 2.2 × 0.8 × 2.1 cm and computed tomography value of about 102 HU were observed behind the L4 vertebral body, with a clear boundary.

CT of the lumbar spine: Bone resorption and destruction changes were observed in the L4 vertebral body and left pedicle; a sparse bone crest structure was noted in the L4 vertebral body, showing palisade-like changes. A mass-like soft tissue shadow with a size of approximately 2.2 × 0.8 × 2.1 cm and a CT value of approximately 102 HU were observed behind the L4 vertebral body, with a clear boundary. The corresponding vertebral canal was compressed and narrowed; its anteroposterior diameter was approximately 7 mm (Fig. 1).

**Figure 2:**
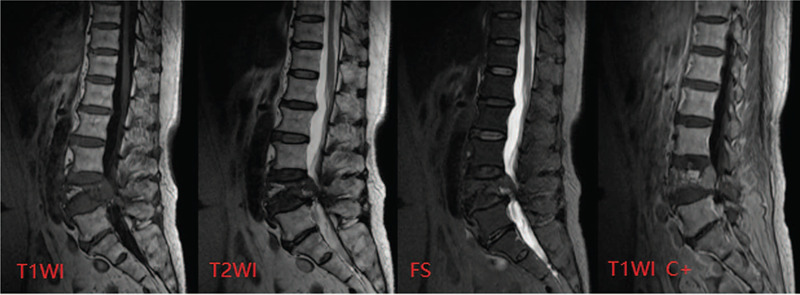
Bone destruction of the L4 vertebral body and left pedicle. An abnormal signal lesion was observed, showing low signal intensity on T1WI, high signal intensity on T2WI, and slightly high signal intensity in the lipid suppression phase. The boundary of the lesion was clear and lobulated. Contrast-enhanced scanning showed slight lesion enhancement. The lesion partially protruded into the spinal canal.

MRI and enhancement of the lumbar spine showed bone destruction of the L4 vertebral body and left pedicle, and an abnormal signal lesion mass was identified, showing low signal intensity on T1WI, high signal intensity on T2WI, and a slightly high signal intensity in the lipid suppression phase. The boundary of the lesion was clear and lobulated. Contrast-enhanced MRI showed that the lesion was slightly enhanced and partially protruded into the spinal canal, with the anterior and posterior diameter of the spinal canal measuring approximately 7 mm (Fig. 2).

**Figure 3:**
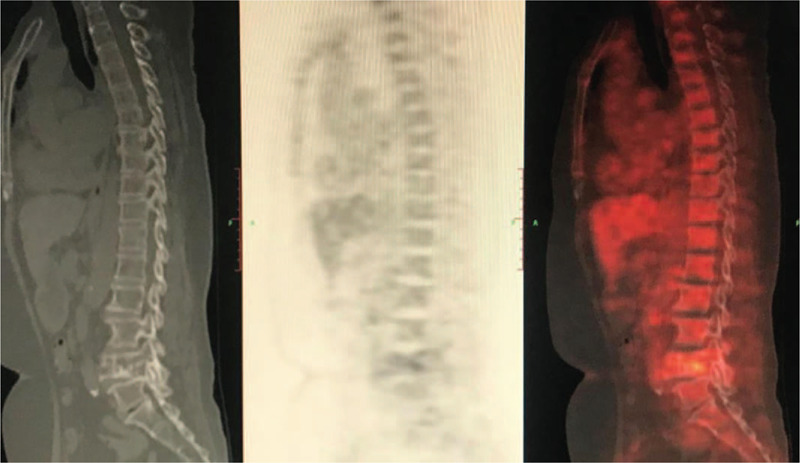
The L4 vertebral body was destroyed by an expansive osteolytic bone with an increased uneven radioactive uptake level. The edge of the destroyed area was hardened, and residual bone ridges were observed in it. The lesion involved the bilateral appendages and locally invaded the spinal canal.

**Figure 4:**
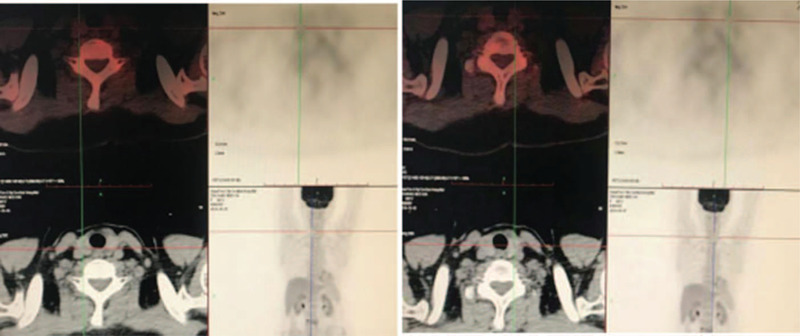
Several nodules with a diameter of about 0.5 to 0.8 cm were observed in the bilateral thyroid lobes. The radioactivity uptake level was not significantly increased.

Positron emission tomography (PET)/CT showed destruction of the L4 vertebral body by an expansive osteolytic bone with an increased uneven radioactive uptake level (SUVmax 7.0), hardening of the edge of the destroyed area, and presence of residual bone ridges. The lesion involved bilateral appendages and locally invaded the spinal canal, with diffuse reductions in the spinal density and hyperosteogeny observed in a majority of the vertebral edges. Several nodules in the bilateral thyroid lobes with a diameter of approximately 0.5 cm to 0.8 cm were observed. The radioactivity uptake level did not significantly increase. No abnormal enlargement or lymph nodes with an abnormal radioactive distribution were found in the bilateral neck. PET/CT of the rest of the trunk and brain showed no obvious abnormal metabolic signs. PET/CT showed:

(1) L4 vertebral body swelling osteolytic bone destruction accompanied by uneven metabolism rate increases; based on the absence of clear signs of malignant tumors in the other parts of the body, the possibility of a metastatic tumor with an unknown primary focus was deemed weak;
(2) Multiple thyroid nodules and low metabolism rate, indicating the possibility of nodular goiter;
(3) PET/CT of the rest of the trunk and brain showed no obvious abnormal metabolic signs (Figs. 3 and 4).

### 2.3 L4 vertebral body surgical process and postoperative pathology

#### 2.3.1 Surgical procedure

Surgery was performed through a left inverted splayed incision, with the patient placed in the right supine position. The skin, subcutaneous, external oblique, internal oblique, and transverse abdominal muscles were cut, layer by layer, from about 4 cm above the iliac crest to the outer edge of the pubic symphysis rectus abdominis, and the peritoneum was cut inward to expose and protect the abdominal aorta. The L4 vertebral body and L3/4 and L4/5 intervertebral discs were exposed, and the left segmental artery was ligated. The upper and lower intervertebral discs of the diseased vertebral body were removed surgically, and the diseased vertebral body and surrounding tissues were gradually separated. Finally, the L4 vertebral body and pathological tissues were removed and complete hemostasis was performed.

#### 2.3.2 Pathology

**Figure 5:**
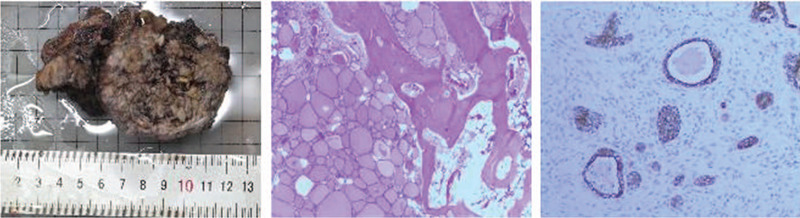
Under a light microscope, some of the bone marrow cavity and cortical bone structures showed destruction. Thyroid follicles were seen as lamellar hyperplasia, with different follicle sizes. The epithelial layer was flat or cubic. The cell morphology was mild, showing no differences. The local follicular nuclei were large, irregular in shape, and disordered in arrangement.

Considering the patient's clinical data and histological morphology, single germ layer teratoma (goiter) with focal follicular epithelial dysplasia was considered, excluding the possibility of thyroid cancer metastasis. Further clinical examination of the thyroid was recommended (Fig. 5)

Immunohistochemistry yielded the following results: galectin-3 (focal+), thyroid transcription factor (TTF-1) (+), CD56 (local-), CK19 (local medium+), human bone marrow endothelium marker-1 (+<5%), Ki67 (+), TPO (focal-), and thyroglobulin (+), indicating the possibility of ectopic goiter.

### 2.4 Thyroid surgery and postoperative pathology

#### 2.4.1 Surgical procedure

After successful anesthesia, the patient was placed in the supine position. The tissue was cut in layers until the bilateral thyroid gland was exposed. An ultrasonic scalpel was used to coagulate and close the bilateral middle vein of the thyroid gland. The glandular lobe was lifted inwards. The upper and lower poles of the thyroid gland and bilateral superior and inferior arteries and veins of the thyroid gland were separated along the lateral edge. The bilateral recurrent laryngeal nerve was fully exposed and protected. A small amount of thyroid tissue at the region from which the bilateral recurrent laryngeal nerve enters the larynx was reserved. The bilateral lobe and thyroid isthmus were completely removed.

#### 2.4.2 Postoperative pathology

**Figure 6:**
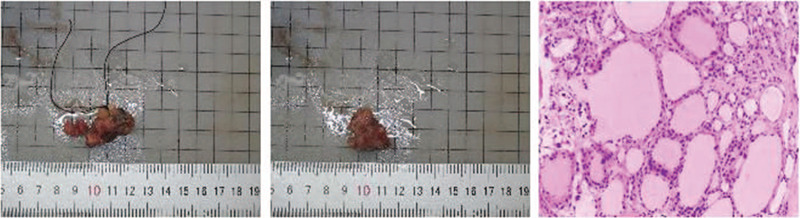
Under a light microscope, the thyroid follicles showed nodular hyperplasia. In some nodules, colloid-like small follicles and normal-size thyroid follicles were mixed and distributed, surrounded by thin layers of fibrous tissue. In addition, we observed interstitial foam cells, multinucleated giant cell hyperplasia, and hemosiderin deposition.

(1) Right thyroid gland: nodular goiter with partial adenomatous hyperplasia with 1 parathyroid tissue;
(2) Left thyroid gland: nodular goiter with an atypical adenomatous hyperplasia nodule;
(3) isthmus thyroid gland: nodular goiter (Fig. 6).

Immunohistochemistry showed: CD56(+), CD19 (local+), galectin-3 (-), human bone marrow endothelium marker-1 (local+), Ki67 (scattered+), TPO (local-), P53 (local weak+), and P16 (local+).

Detection of BRAF V600E gene protein: BRAF V600E (—)

(Note: The protein was detected using the VENTANA BenchMark XT automatic detection system with the analyzer/VENTANA anti-BRAF V600E antibody and VENTANA OptiView DAB IHC detection kit.)

Based on the combination of the above-mentioned pathological results pertaining to the lumbar pathological tissues and thyroid gland, the possibility of metastatic thyroid cancer was excluded. Pathology of the vertebral body tissues led to the diagnosis of ectopic goiter.

## 3 Discussion

The thyroid gland is an endocrine gland comprising 2 lateral lobes connected by the thyroid isthmus. Ectopic thyroid is usually associated with extensive morphological variations and dysplasia. At a gestational age of 4 weeks, the thyroid gland starts to develop mainly through the invagination of primitive pharyngeal ventral bottom endoderm cells.^[1]^

Ectopic thyroid is the most commonly observed form of thyroid dysplasia, accounting for 48% to 61% of all such cases. Ectopic thyroid is predominantly diagnosed before age 30 years, and is more commonly reported in women.^[3]^ In this article, we presented the case of a 67-year-old woman with a lumbar ectopic goiter. This is the first case report of its kind in this context.

Ectopic goiter is diagnosed using radionuclide thyroid imaging, ultrasound, CT, nuclear MRI, biopsy, and thyroid function tests. CT shows greater efficacy in the evaluation of bone differentiation, while MRI is more suited to the evaluation of soft-tissue differentiation. Radionuclide thyroid imaging is based on isotope uptake and usually reflects a lesion's metabolic rate. However, due to the low prevalence of diseases, CT, radionuclide thyroid imaging, and nuclear MRI are associated with high rates of misdiagnosis.^[4]^ The CT value of the ectopic thyroid tissue in our patient was 104 HU on plain CT, showing high density. Compared to the findings in the surrounding muscle tissue, MRI showed low signal intensity on T1WI, high signal intensity on T2WI, a slightly high signal intensity in the lipid suppression phase, and slight mass enhancement on enhanced scanning. PET/CT demonstrated expansive osteolytic destruction of the L4 vertebral body with an increased uneven radioactive uptake level. Thus, it can be considered that ectopic goiters have no specific manifestations on radionuclide thyroid imaging, CT, and nuclear MRI, owing to which reaching a diagnosis using these methods is very difficult. Biopsy is still required for the provision of a final diagnosis.

TTF-1 has been shown to be related to thyroid gene abnormalities.^[5,6]^ Previous studies have shown that the expression of TTF-1 in the ectopic thyroid gland is stronger than that in the in situ thyroid gland. Therefore, abnormal TTF-1 expression may play a role in ectopic thyroid development. In addition, increased TTF-1 expression levels may lead to dysfunction, as observed in many ectopic thyroid patients.^[7]^ In our patient, TTF-1-positivity was observed. Thyroglobulin was only synthesized in thyroid tissue, and its expression was positive in this case. However, previous studies have shown that ectopic thyroid can participate in thyroid hormone biosynthesis, even if the amount of thyroid hormone is insufficient.^[7]^ Ki-67-positive tumor cells are usually associated with malignant transformation; however, no previous studies have reported on the expression of Ki-67 in ectopic thyroid tissue.^[7]^ In our patient, the expression of Ki-67 in the ectopic goiter was relatively low.

While asymptomatic patients with an ectopic thyroid usually do not require treatment, there is a need for observation. For symptomatic patients, treatment depends on gland size, the nature of the symptoms, thyroid function, and histological findings.^[3]^ At present, there is no consensus on the best treatment strategy for ectopic thyroid, probably owing to the low prevalence of the condition in clinical settings. In this case, the diseased tissue of the patient damaged the L4 vertebral body and left pedicle bone and protruded into the vertebral canal, resulting in spinal canal stenosis. This led to a series of clinical symptoms, including waist and leg pain and aggravation. Treatment in such cases is usually surgical resection for the provision of relief from clinical symptoms.

Generally, the differential diagnoses of ectopic thyroid tissue on imaging include metastatic cancer, bone-derived tumor, non-tumor tissue, thyroid tumor, and teratoma. It is important to determine whether the thyroid tissue of the diseased vertebral body is an ectopic goiter or thyroid papillary carcinoma or follicular carcinoma metastasis. Therefore, we performed thyroidectomy and thyroid histopathological examination according to the patient's condition. Immunohistochemical staining and molecular detection were used for the achievement of a final diagnosis. We found the absence of cancerous tissue in the patient's thyroid tissue, eliminating the possibility of thyroid cancer metastasis.

## 4 Conclusion

Clinically, the final diagnosis of ectopic goiter is reached through the use of radionuclide thyroid imaging, ultrasound examination, CT, nuclear MRI, and biopsy pathology. However, the imaging manifestations in this case were atypical, leading to difficulties in reaching a diagnosis. Conclusions were finally drawn through pathology.

## Acknowledgments

We would like to thank Editage (www.editage.com) for English language editing.

## Author contributions

**Conceptualization:** Qihuan Lin, Wenbin Zheng.

**Data curation:** Qihuan Lin, Wenbin Zheng.

**Investigation:** Rong Fan, Li Zhang, Qilu Gao.

**Methodology:** Hongkun Liu, Weijia Li, Qilu Gao.

**Resources:** Qihuan Lin, Wenbin Zheng.

**Writing – original draft:** Qihuan Lin, Wenbin Zheng.

## Abbreviations

Abbreviations: CT = computed tomography, MRI = magnetic resonance imaging, TE = echo time, TPO = thyroid peroxidase, TR = repetition time, TTF-1 = thyroid transcription factor 1.
